# Supplementary material for: Comparative Genome-Wide Analysis of Two Caryopteris x Clandonensis Cultivars: Insights on the Biosynthesis of Volatile Terpenoids
Source: Plants (Basel). 2023 Feb 1;12(3):632. doi: 10.3390/plants12030632 (PMC9921992; doi:10.3390/plants12030632)
Supplement: Supplementary file 1 [file plants-12-00632-s001.zip › Supplemental_Ritz.pdf]

Supplementary figures

Within these supplementary data following figures can be found:

Figure S1. Chemical structure of limonene backbone and difference to C6-C4-shift in α-pinene

Figure S2. Cultivars of *Caryopteris x clandonensis* used in this manuscript. (A) Cultivar Good as Gold (B) Cultivar Pink Perfection was grown in soil as displayed (C) Comparison of plant leaves: Pink Perfection, Hint of Gold, Good as Gold, Dark Knight (l.t.r.)

Figure S3. PacBio sequencing quality reports of different *Caryopteris x clandonensis* cultivars. (A) Cultivar Dark Knight, read length distribution of Hifi reads. High molecular weight genomic DNA was cleaned using AmPureBeads protocol step (B) Cultivar Dark Knight, Comparison between processed read length and polymerase read length. (C) Cultivar Pink Perfection, read length distribution of Hifi reads. High molecular weight genomic DNA was cleaned using AmPureBeads protocol step (D) Cultivar Pink Perfection, Comparison between processed read length and polymerase read length.

Figure S4. GenomeScope profile of k-mer analysis of Dark Knight sequencing reads. k indicates the size of kmers used, aa indicates the level of heterozygosity and p the estimated ploidy. Len estimates the genome size including ploidy level.

Figure S5. GenomeScope profile of k-mer analysis of Pink Perfection sequencing reads. k indicates the size of kmers used, aa indicates the level of heterozygosity and p the estimated ploidy. Len estimates the genome size including ploidy level.

Figure S6. Synteny evaluation between the *Caryopteris x clandonensis* cultivars Dark Knight (bottom: + and – strand) and Pink Perfection (top: based as reference). Shown in colored boxes are the clusters which are similar between both cultivars. Links show the position of synteny on both genomes relative to each other.

Chemical structure of D-limonene and α-pinene

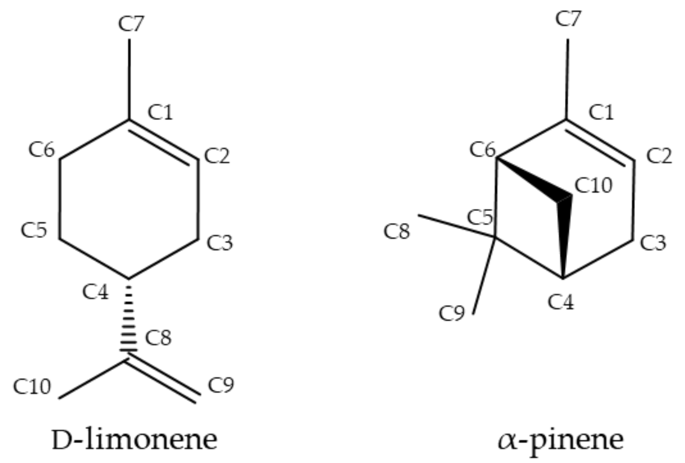

Figure S 1. Chemical structure of D-limonene backbone and difference to C6-C4 shift in α-pinene

Plant cultivars

A B

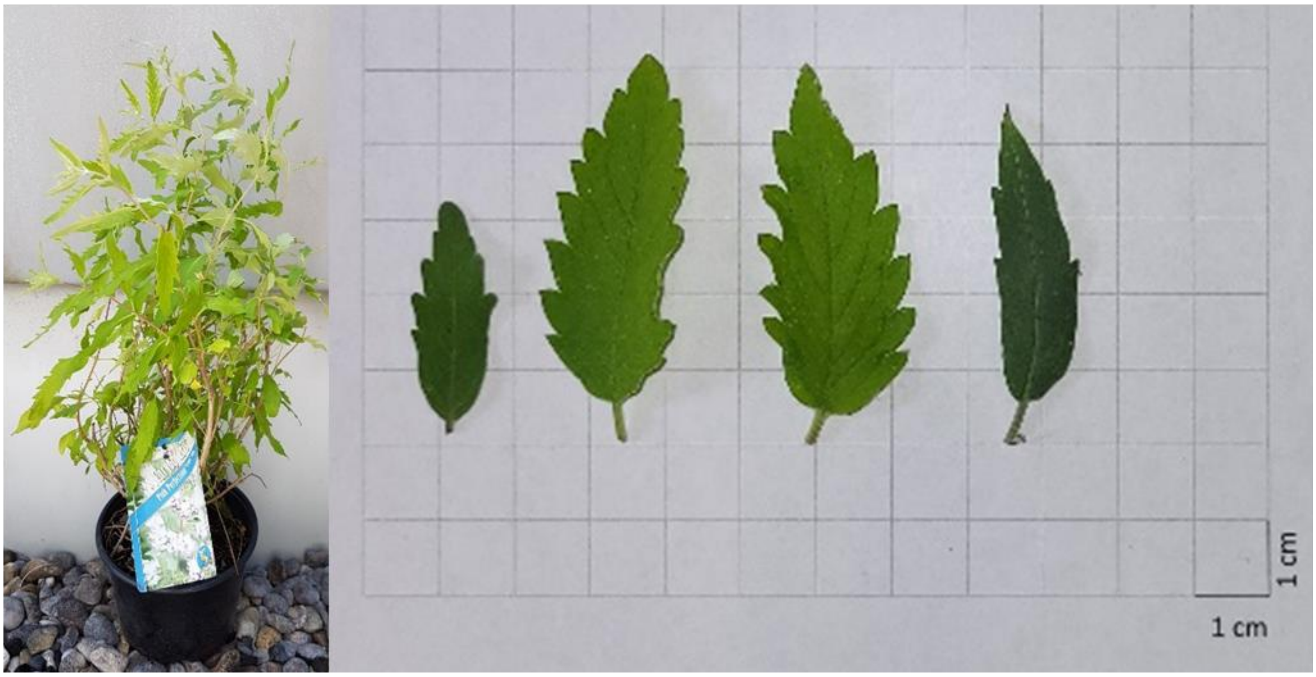

Figure S2. Cultivars of *Caryopteris x clandonensis* used in this manuscript. (A) Cultivar Pink Perfection was grown in soil as displayed (B) Comparison of plant leaves: Pink Perfection, Hint of Gold, Good as Gold, Dark Knight (l.t.r.)

PacBio sequencing quality reports

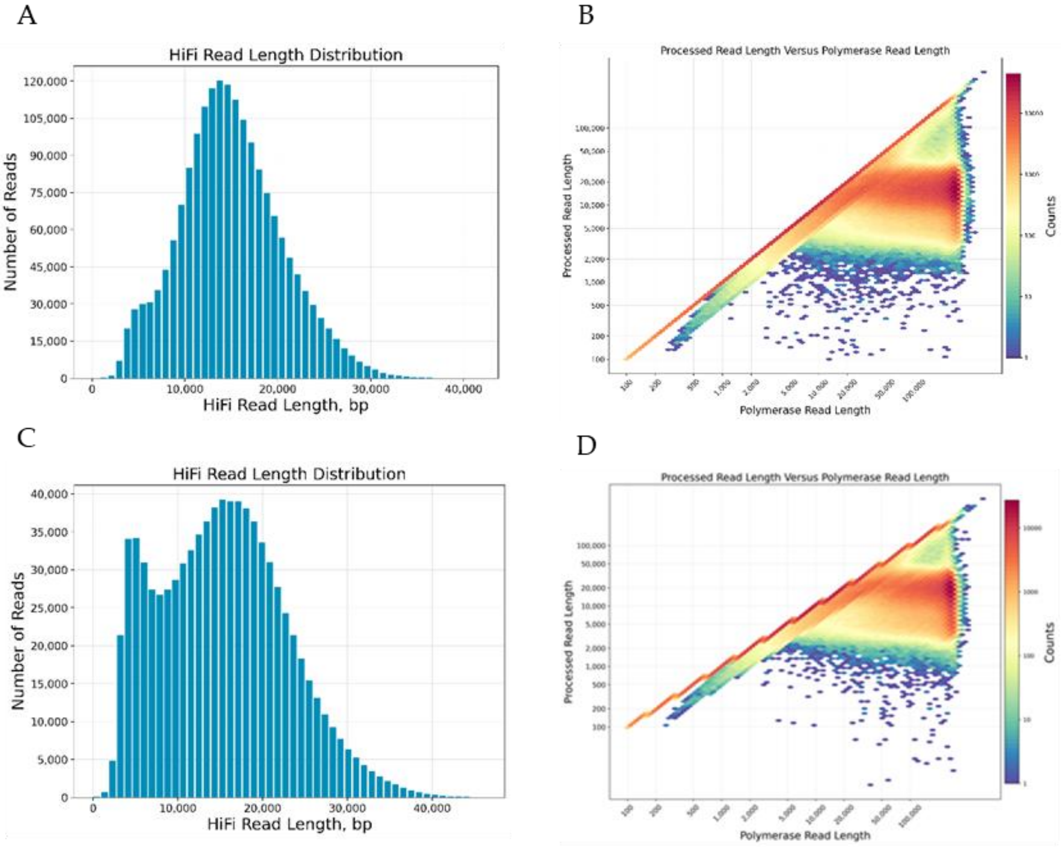

Figure S3. PacBio sequencing quality reports of different *Caryopteris x clandonensis* cultivars. (A) Cultivar Dark Knight, read length distribution of Hifi reads. High molecular weight genomic DNA was cleaned using AmPureBeads protocol step (B) Cultivar Dark Knight, Comparison between processed read length and polymerase read length. (C) Cultivar Pink Perfection, read length distribution of Hifi reads. High molecular weight genomic DNA was cleaned using AmPureBeads protocol step (D) Cultivar Pink Perfection, Comparison between processed read length and polymerase read length.

K-mer based analysis of PacBio long reads on Dark Knight and Pink Perfection

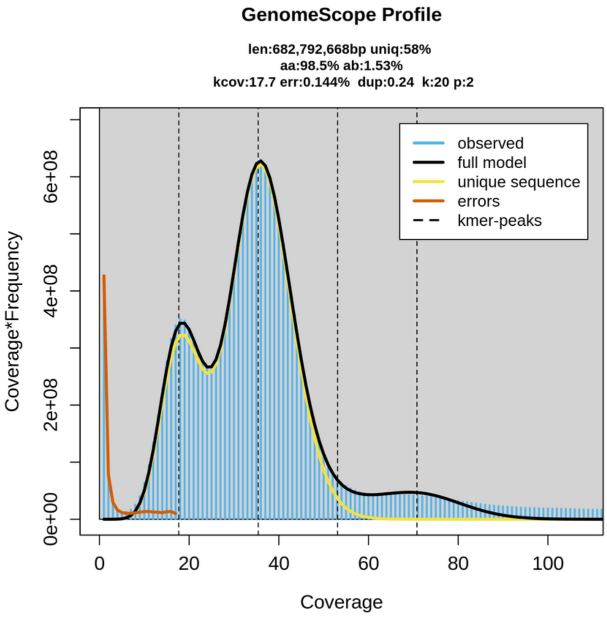

Figure S4. GenomeScope profile of k-mer analysis of Dark Knight sequencing reads. k indicates the size of k-mers used, aa indicates the level of heterozygosity and p the estimated ploidy. Len estimates the genome size including ploidy level.

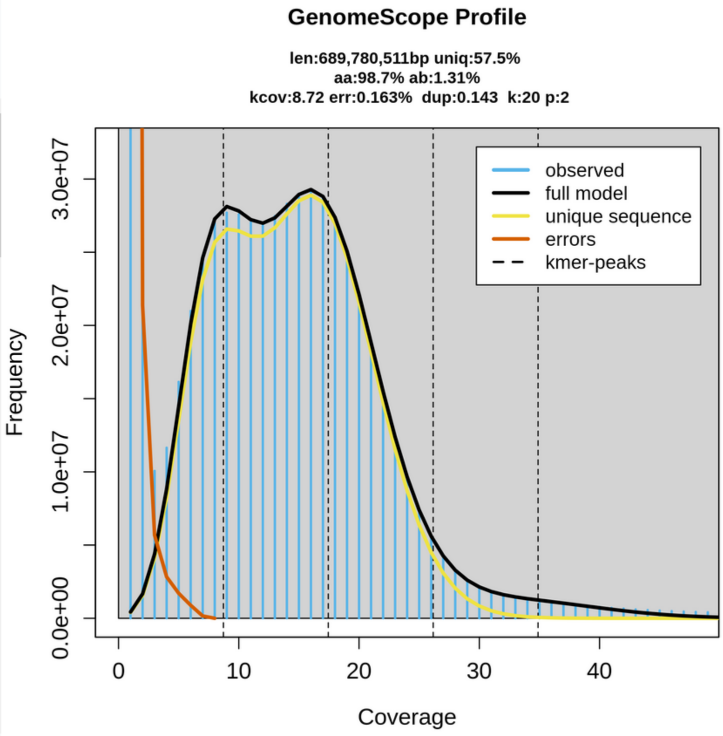

Figure S5. GenomeScope profile of k-mer analysis of Pink Perfection sequencing reads. k indicates the size of k-mers used, aa indicates the level of heterozygosity and p the estimated ploidy. Len estimates the genome size including ploidy level.

Synteny evaluation of Dark Knight (bottom two lanes) and Pink Perfection (upper lane)

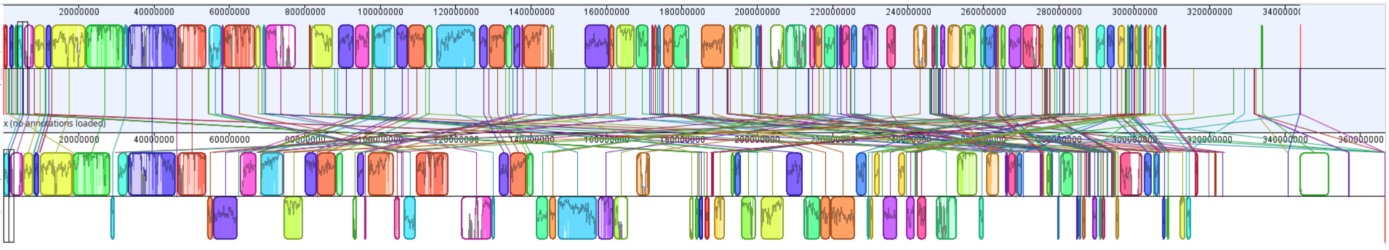

Figure S6. Synteny evaluation between the *Caryopteris x clandonensis* cultivars Dark Knight (bottom: + and – strand) and Pink Perfection (top: based as reference). Shown in colored boxes are the clusters which are similar between both cultivars. Links show the position of synteny on both genomes relative to each other.
